# A pan-cancer analysis of the prognostic implication and oncogenic role of tubulin epsilon and delta complex 2 (TEDC2) in human tumors

**DOI:** 10.3389/fimmu.2023.1272108

**Published:** 2024-01-04

**Authors:** Yang Liu, Jie Zhu, Jing Shen, Yuting Lu, Ke Pan, Chuan Tong, Yao Wang

**Affiliations:** ^1^ Faculty of Hepato-Pancreato-Biliary Surgery, the First Medical Centre, Chinese PLA General Hospital, Beijing, China; ^2^ Senior Departments of Urology, the Third Medical Centre, Chinese PLA General Hospital, Beijing, China; ^3^ Department of Endocrinology, the Eighth Medical Center of PLA General Hospital, Beijing, China; ^4^ Department of Bio-therapeutic, the First Medical Center, Chinese PLA General Hospital, Beijing, China

**Keywords:** immune infiltration, tumor microenvironment, pan-cancer, prognosis, TEDC2

## Abstract

**Introduction:**

Tubulin epsilon and delta complex 2 (TEDC2) is widely expressed in various human tissues and primarily governs centriole stability. However, the biological significance of TEDC2 in pan-cancer is unclear.

**Methods:**

In this study, we employed R software and various online bioinformatics analysis tools to investigate the functional attributes of TEDC2 in human tumours and its potential involvement in immune response. The status of TEDC2 expression was evaluated in samples from the TCGA and GEO datasets, as well as in tumour and corresponding normal samples from the TCGA database. Subsequently, Kaplan-Meier estimates, clinical correlations, and univariate Cox regressions were used to analyze the 33 types of tumors from TCGA and determine the prognostic significance of TEDC2. Moreover, nomogram models were formulated using three distinct tumours, namely kidney renal clear cell carcinoma (KIRC), lung adenocarcinoma (LUAD), and liver hepatocellular carcinoma (LIHC), to evaluate the prognostic significance of TEDC2 in tumours. Furthermore, TEDC2 was investigated for its correlation with the levels of immune cell infiltration, and a functional enrichment analysis was conducted to identify potential signalling pathways involving TEDC2.

**Results:**

Differential analysis revealed that 16 tumour types expressed TEDC2 to a greater extent than normal tissues. The abnormal expression of TEDC2 can predict survival outcomes in patients with adrenocortical carcinoma (ACC), KIRC, kidney renal papillary cell carcinoma (KIRP), LUAD, LIHC, lower grade glioma (LGG), and thymoma (THYM). Subsequent results indicated that TEDC2 has the ability to influence ECM regulators, cell cycle, and Immune checkpoint-associated signalling pathways, which could potentially lead to a poor prognosis and tumour progression.

**Discussion:**

TEDC2 has been identified as a potential therapeutic target that could predict the prognosis of multiple tumour types, making it a promising target for reversing tumour development.

## Introduction

Globally, tumors pose a serious threat to public health, with incidence and mortality rates on the rise (1, 2). Despite significant advancements in tumor diagnosis and treatment, the 5-year overall survival rate for most tumors remains dismal (3). Therefore, there is an urgent need for novel approaches to diagnose and treat tumors. Currently, the utilization of tumor biomarkers has greatly enhanced the prognosis in certain types of tumors (4–6).

The rapid advancements in next-generation sequencing and bioinformatics have facilitated the accumulation of data, enabling a comprehensive understanding of the intricate biological characteristics of tumors from various perspectives. Concurrently, a growing number of databases, such as the Gene Expression Omnibus (GEO) and The Tumor Genome Atlas (TCGA), have been established to comprehensively analyze the pathogenesis of cancer. These databases have conducted molecular characterizations on more than 20,000 primary tumors and their corresponding normal samples, encompassing 33 different types of cancer. In a recent study, Pan et al. employed a pan cancer analysis approach to investigate the impact of abnormal expression of the RUNX gene on the prognosis of diverse tumors (7). This analysis involved the utilization of TCGA multi-omics data in conjunction with various online tools. Similarly, Xie et al. developed a “FOXOs score” system based on the TCGA database, which demonstrated a correlation with multiple immune features and the ability to accurately predict treatment efficacy across various GEO datasets (8). Consequently, the utilization of these extensive and multi-omics tumor datasets can serve as an effective means of identifying potential tumor biomarkers.

Tubulin epsilon and delta complex 2 (TEDC2), also named Chromosome 16 open reading frame 59 (C16orf59), is a protein coding gene. Some studies reported that TEDC2 is involved in the regulation of centriole stability, ciliary hedgehog signaling, and might contribute to the tumorigenesis of LUAD (9, 10) and central nervous system lymphoma (11), but no comprehensive study have been conducted on the immune characteristics and prognostic of TEDC2 in tumors. Furthermore, Meng et al. employed the monozygotic twin-pair database to identify alterations in DNA methylation subsequent to alcohol consumption (12). Their findings revealed a significant correlation between elevated methylation levels of cg07326074, situated within the TEDC2 gene, and alcohol intake. It is worth noting that prolonged alcohol consumption has been associated with immune dysfunction in the body (13), and it is widely recognized as a prominent risk factor in the development of diverse tumors (14–16). The observed methylation patterns linked to alcohol consumption are hypothesized to impact the functionality of the TEDC2 gene. Currently, there are no reports on the role of TEDC2 in pan-cancer. Therefore, we investigated the mechanisms of TEDC2 in tumors and its correlation with immune infiltration. In our study, we found that the expression of TEDC2 was unregulated in the majority of tumors, thereby affecting the prognosis of ACC, KIRC, KIRP, LUSC, LIHC, and MESO. According to immune infiltration analysis, TEDC2 expression was associated with multiple immune cells, and might affect tumor survival. Furthermore, enrichment analysis indicated that TEDC2 may be involved in the tumorigenesis by the cell cycle, ECM regulators, and Immune checkpoint-associated signaling pathways. Collectively, these findings indicate that TEDC2 plays multifaceted roles across tumors, can influence the prognosis and immune infiltration of some tumors, and could become a novel biomarker.

## Materials and methods

### Data acquisition

Expression profile data for 33 tumors and corresponding clinical data were obtained from The Cancer Genome Atlas (TCGA, https://portal.gdc.cancer.gov/). Additionally, the RNA-seq data of GSE10927 (10 normal tissues, 55 tumorous tissues), GSE15641 (23 normal tissues, 69 tumorous tissues), GSE36376 (193 normal tissues, 240 tumorous tissues), GSE51575 (26 normal tissues, 26 tumorous tissues), GSE63514 (24 normal tissues, 28 tumorous tissues), GSE116959 (11 normal tissues, 57 tumorous tissues), GSE13213 (117 tumorous tissues), GSE3141 (111 tumorous tissues), GSE214992 (32 cell line samples) and GSE91061 (37 tumorous tissues) were downloaded from Gene Expression Omnibus (GEO, https://www.ncbi.nlm.nih.gov/). The 100 genes most closely related to TEDC2 were obtained from The Gene Expression Profiling Interactive Analysis database (GEPIA2, http://gepia2.tumor-pku.cn/#index). The protein–protein interaction (PPI) network was analyzed in STRING (https://cn.string-db.org/).

### Expression analysis of TEDC2

The mRNA expression levels of TEDC2 in normal tissues and tumors were analyzed and visualized using the ggplot2 package (version 3.4.2). The representative immunohistochemical results of TEDC2 in tumor tissues were obtained from the Human Protein Atlas (HPA, https://www.proteinatlas.org/).

### Diagnostic and prognostic value of TEDC2

Kaplan−Meier survival analysis was employed to assess the association between TEDC2 expression and clinical outcomes, including overall survival (OS), disease specific survival (DSS), and progression free interval (PFI) in TCGA datasets. In addition, a receiver operating characteristic curve (ROC) was drawn for tumors in which TEDC2 affects prognosis. The ggplot2 package (version 3.4.2) was used to analyze and visualize the correlation between TEDC2 expression and multiple clinical parameters such as age, gender and pathologic stage.

Univariate Cox regression analysis of factors related to OS was performed for tumors in which TEDC2 affects prognosis. Tumors with p < 0.05 and three representative tumors (KIRC, LUAD, LIHC) were selected as the training set for constructing a nomogram model. Calibration curves were generated to assess the prediction accuracy of the nomograms at 1, 3, and 5 years.

### Genetic alteration analysis

cBioPortal database (https://www.cbioportal.org/) (17, 18) was used to estimate TEDC2 genetic alterations in tumors using data from the TCGA Pan-Cancer Atlas Studies. According to the data set of TCGA Pan-Cancer Atras Studies, we calculated the mutation frequency and copy number change of TEDC2 gene in the “Cancer Type Summary” module. A mutation site plot of TEDC2 was created using the “Mutations” module.

To analyze the correlation between TEDC2 mutation status and SKCM, BRCA, and UCES prognosis, the molecular profile was selected as mutations based on “skin cutaneous melanoma (TCGA Pan-Cancer)”, “breast invasive carcinoma (TCGA Pan-Cancer)”, “uterine corpus endometrial carcinoma (TCGA Pan-Cancer)”, and the survival plot was generated by dividing cases into altered and unaltered groups.

### Immune infiltration analysis

The GSVA package (version 1.48.0) was used to perform Spearman correlation analysis of TEDC2 expression and immune cell infiltration, including activated DC (aDC), DC, immature DC (iDC), plasmacytoid DC (pDC), macrophages, mast cells, neutrophils, eosinophils, cytotoxic cells, B cells, NK cells, NK CD56bright cells, NK CD56dim cells, T cells, CD8 T cells, T central memory (Tcm), T effector memory (Tem), T helper cells, T gamma delta (Tgd), T follicular helper (Tfh), Th1 cells, Th2 cells, Th17 cells and Treg (19, 20).

### Functional enrichment analysis and PPI network analysis

The GEPIA2 database was used to obtain the 100 genes most closely related to TEDC2. We performed Gene Ontology (GO) analysis, which includes biological pathway (BP), and molecular function (MF) and cellular component (CC) categories. We also performed Kyoto Encyclopedia of Genes and Genomes (KEGG) analysis based on the TEDC2 related genes to further explore the potential functions of TEDC2. Additionally, a PPI network of the 100 TEDC2 related genes was created from the STRING database (21), and the top 10 molecules were extracted by the cytoHubba plugin in the Cytoscape (version 3.7.1) software.

### Differential expression analysis and gene set enrichment analysis

Samples were divided into high and low groups based on the median expression level of TEDC2. DESeq2 package (version 1.40.1) was used to analyze differential expression of TEDC2 in tumors in which can affect prognosis. Using | log2 (FC) |>2 and p.adj<0.01 as conditions for screening significantly different genes. Then, according to the results obtained from the differential expression analysis of TEDC2 in different tumors, GSEA was performed using the clusterProfiler package (version 4.8.0) (22, 23). Subsequently, these genes were enriched on the basis of the Hallmark gene sets database. Gene sets with normalized enrichment score (NES) > 1, and false discovery rate (FDR) < 0.05 were considered significant results.

### Cell culture and transfection

Human normal liver cell line L02, human LUAD cell line A549 and human LIHC cell line HepG2 were obtained from the Cell Bank of the Chinese Academy of Sciences and cultured in Dulbecco’s modified Eagle’s medium (DMEM, Hyclone, USA) supplemented with 10% Fetal bovine serum (FBS, Gibco) and 100 units/mL penicillin at 37°C with 5% CO2. The small interfering RNAs (siRNAs) targeting human TEDC2 and a negative control were purchased from Shanghai Genechem Co., Ltd. The sequence of TEDC2 siRNA (siTEDC2) were 5’- GCGCACAGCGACAATTGCAATTGGA-3’, 5’- GCCAGAAACTAATGGAGAGGA-3’ and the sequence negative control siRNA (siNC) was 5’- GCGGACAGCAACGTTAACTTCAGGA-3’. Transient transfections were conducted following the manufacturer’s protocol (Entranster-R4000, Engreen Biosystem). A549 and HepG2 cells were seeded in 12 well plates one day prior to transfection and were transfected when the cell confluence reached 40%. The culture medium was replaced with fresh medium after 6 hours of transfection. Finally, cells were harvested for further experiments after 24 hours of transfection.

### Cell proliferation assays

To ascertain cellular proliferation, a quantity of 1 × 10^4^ cells was introduced into a 24 well plate. The cells were cultivated in DMEM supplemented with 10% Fetal bovine serum, with the medium being refreshed on a daily basis. Subsequently, at 24, 48, 72 and 96 hour intervals, the cells were harvested, diluted with a trypan blue working solution, and enumerated using an automatic cell counter Arthur (NanoEntek, Germany) to establish a growth curve. Each measurement was performed in triplicate, and a minimum of three independent experiments were conducted.

### Real-time PCR

The SYBR Premix Ex Taq was employed for the purpose of gene mRNA expression detection in various cell types through the utilization of real-time PCR on the ABI7500 instrument. In order to ensure consistency, three tests were conducted on each sample, with β-Actin serving as the standardization control. The relative mRNA concentration was determined by averaging the results of the three replicates, and the 2^-△△CT^ method was employed to calculate the expression levels. The specific primer sequence can be found in **Supplementary Table 1**.

### Cell cycle assay

4 × 10^5^ harvested cells were incubated in phosphate buffered saline (PBS, Hyclone, USA) containing 0.1% Triton X-100 (Sigma, USA) and 0.2 mg/mL RNaseA (Sigma, USA), followed by fixation in 75% alcohol at 4°C for 60 minutes. After three washes with cold PBS, 7-amino dactinomycin (7-AAD, BD, USA) was added and incubated at 37°C for 30 minutes. Subsequently, cell cycle analysis was performed using flow cytometry (BECKMAN COULTER, USA). Each measurement was repeated three times, and a minimum of three experiments were conducted.

### Wound healing assay

A density of 4 × 10^5^ cells was inoculated into each well of a 6 well plate. Following overnight incubation, the cell monolayer was scraped using sterile pipette tips. The floating cells were then washed with PBS and cultured with DMEM. Migration images along the scratch line were captured at intervals of 0, 6, and 12 hours using an optical microscope. The measurement of wound area was conducted using Image J software from the National Institutes of Health in Bethesda, USA. The migration rate (%) was calculated as ((A - B)/A) × 100%, where A represents the wound area at 0 hours and B represents the wound area at 6 and 12 hours. The experiments were conducted in triplicates independently.

### Transwell assay

Cell migration and invasion experiments were conducted using a 24 well plate with an 8 µm pore chamber (Corning, USA). For the invasion experiment, the upper chamber of the Transwell pore chamber was coated with a 1:8 dilution of Matrigel matrix gel (BD, USA). Prior to experimentation, cells were cultured in DMEM medium without FBS for 12 hours to induce starvation treatment. Subsequently, the cells were suspended in DMEM medium without FBS and added to the upper chamber at a concentration of 1 × 10^5^ cells per well. Simultaneously, DMEM medium containing 10% FBS was added to the lower chamber, and the plate was incubated in an incubator for a duration of different time points. Following incubation, the residual cells adhered to the filter membrane surface should be delicately removed using a cotton swab. Subsequently, the cells that migrated to the lower surface of the filter membrane ought to be fixed with methanol for a duration of 20 minutes, followed by staining with a 0.1% Crystal violet solution for the same duration. To ensure accuracy, the microscope should be inverted to observe the lower surface and the counting process should be repeated three times. It is important to note that the steps involved in the cell migration experiment closely resemble those of the invasion experiment, with the exception that no gel coating is applied.

### Statistical analysis

Spearman rank test and Wilcoxon rank-sum test were respectively performed to examine correlation between two groups and the expression difference. Log-rank test was used to compare survival differences between groups. Univariate and multivariate Cox proportional hazard regression analyses were performed to screen the factors influencing the prognosis. Statistical analyses were performed using GraphPad Prism 9 and R (version 4.3) software. P values < 0.05 were considered statistically significant. (*p < 0.05, **p < 0.01, ***p < 0.001, and ****p < 0.0001)

## Results

### The expression of TEDC2 across tumors

**Figure 1:**
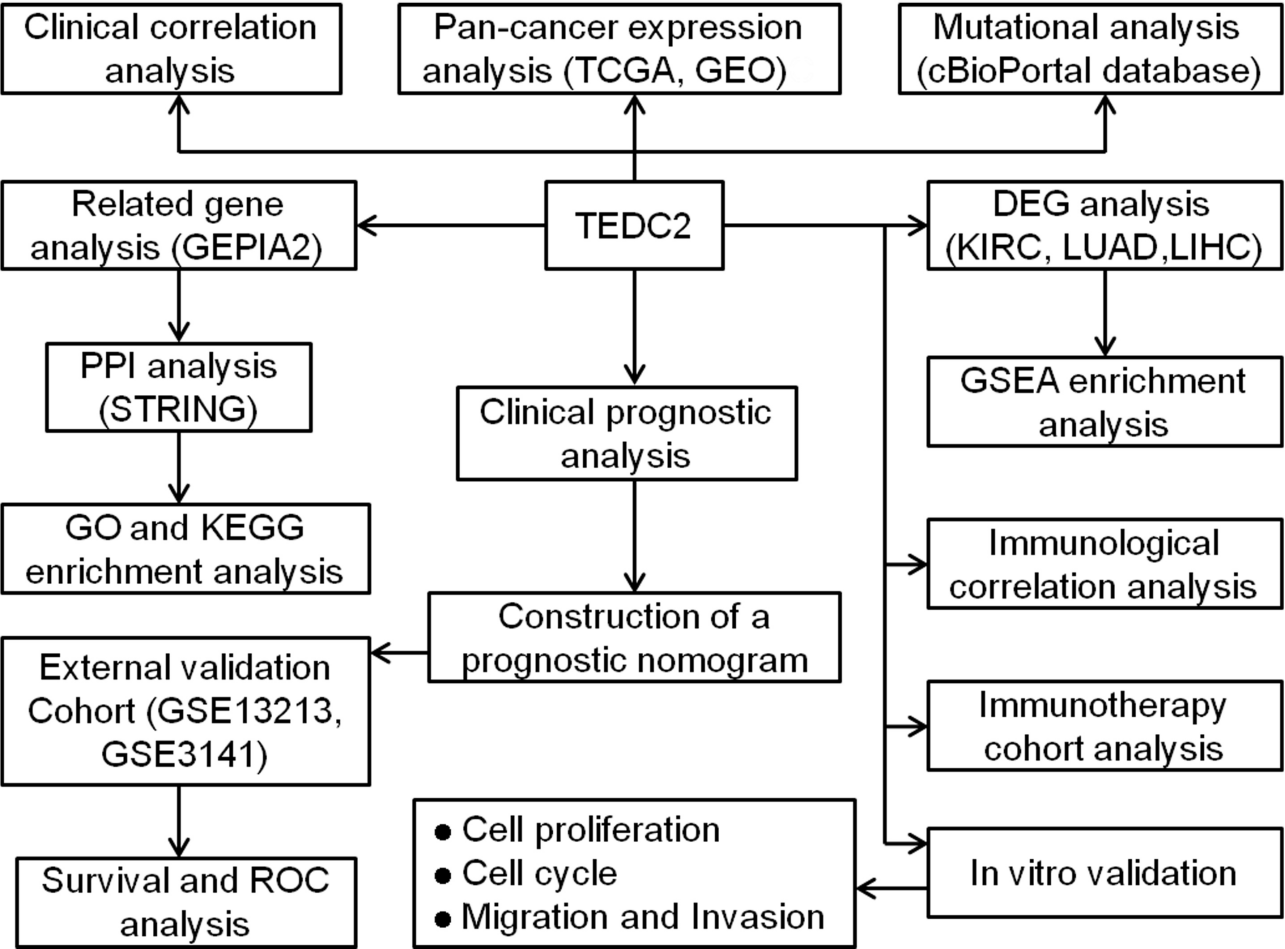
Workflow diagram of this study.

**Figure 2:**
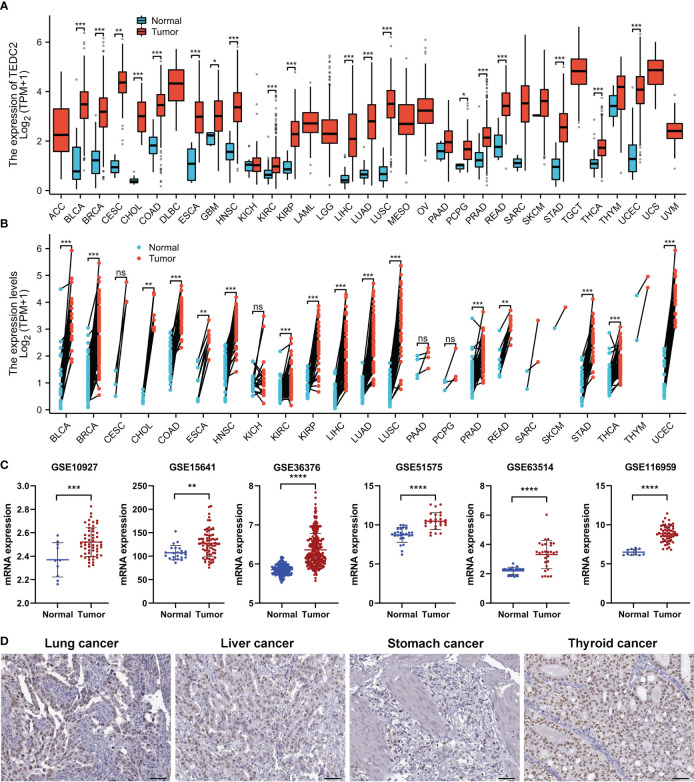
The mRNA expression of TEDC2 in pan-cancer. **(A)** TEDC2 expression in 33 tumors in TCGA database. **(B)** TEDC2 expression in paired samples of 22 tumors in TCGA database. **(C)** TEDC2 expression in the six GEO database. **(D)** The IHC images of TEDC2 in tumor tissues extracted from the HPA. (ns, *p < 0.05, **p < 0.01, ***p < 0.001, and ****p < 0.0001).

The workflow in the current study is demonstrated in **Figure 1**. The aberrant expression of genes in tumor samples was related to probably participate in tumorigenesis. To clarify the expression of TEDC2 across tumors, normalized TCGA data were analyzed. The results showed that TEDC2 was significantly upregulated in many tumors compared to corresponding normal tissues, including KIRP, KIRC, LIHC, STAD, LUAD and so on (**Figure 2A**). TEDC2 expression was also analyzed in 23 types of tumors and paired normal tissues, and the result was roughly in consistent with the unpaired samples (**Figure 2B**). Furthermore, the differential expression of TEDC2 between tumors and normal tissues was verified by the data sets GSE10927, GSE15641, GSE36376, GSE51575, GSE63514 and GSE116959. The results showed that the expression of TEDC2 in many tumors was higher than normal tissues (**Figure 2C**). In order to substantiate these findings, we conducted an analysis of the immunohistochemistry of TEDC2 across various tumors within the HPA database, results demonstrated that a noteworthy increase in the expression of the TEDC2 protein within certain tumor samples (**Figure 2D**).

### Genetic alteration analysis of TEDC2 across tumors

We observed the genetic alteration status of TEDC2 in different tumor samples of the TCGA cohorts. We found that all cholangiocarcinoma cases with genetic alteration (~3% frequency) had miss mutation of TEDC2, and adrenocortical carcinoma tumor samples had the highest TEDC2 genetic alteration frequency (>4%). It is worth noting that amplification, deep deletion, and miss mutation were the main types of frequent genetic alterations in TEDC2. (**Supplementary Figure 1A**). A total of 76 TEDC2 mutations, including 13 truncating mutations, 57 missense mutations, 4 splice mutation and 2 fusion mutations were detected in TCGA tumor samples (**Supplementary Figure 1B**). In addition, we assessed whether genetic variation of TEDC2 is associated with clinical survival prognoses with different types of tumor. The results showed that the altered TEDC2 did not cause a significant difference in overall survival (**Supplementary Figure 1C**). It is noteworthy that the occurrence of TEDC2 mutation is relatively infrequent in the majority of tumors, thus necessitating additional validation through the inclusion of a larger dataset comprising clinical patient information.

### The association between TEDC2 expression and prognosis across tumors

**Figure 3:**
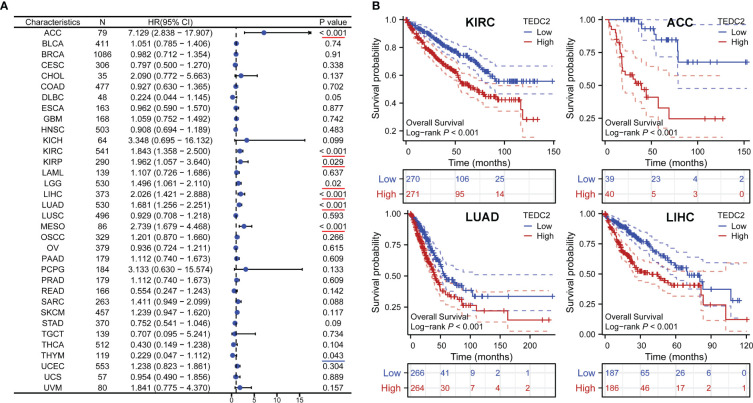
The correlation between TEDC2 expression and OS in pan-cancer. **(A)** Forest plots showed the effect of TEDC2 expression on OS in pan-cancer. The presence of a red underline signifies an unfavorable prognosis for TEDC2, whereas a blue underline denotes a favorable prognosis for TEDC2. **(B)** The effects of TEDC2 expression on OS in KIRC, LUAD, ACC and LIHC, respectively.

To explore the prognostic value of TEDC2 across tumours, Kaplan‒Meier survival analysis was performed to assess the association between TEDC2 expression and clinical outcome. We investigated the association between TEDC2 expression and OS in tumours (**Figure 3A**), and the results showed that high expression of TEDC2 was associated with significantly shorter OS in ACC (HR = 7.129, 95% CI 2.838–17.907, p < 0.001) , KIRC (HR = 1.843, 95% CI 1.358–2.5, p < 0.001), LUAD (HR = 1.681, 95% CI 1.256–2.251, p < 0.001) and LIHC (HR = 2.026, 95% CI 1.421–2.888, p < 0.001) (**Figure 3B**). Subsequently, we investigated the association between TEDC2 expression and DSS and PFI in tumours, which was roughly in agreement with the result of OS (**Supplementary Figure 2**).

### Construction and validation of a nomogram on TEDC2

**Figure 4:**
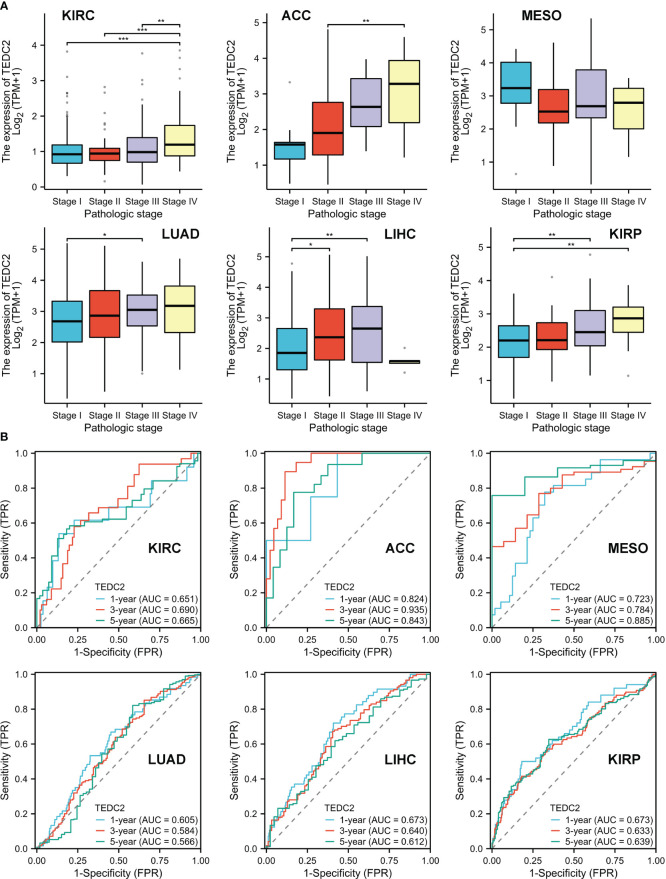
The correlation between TEDC2 expression and clinic pathological parameters. **(A)** The expression of TEDC2 was correlated with pathologic in ACC, KIRC, MESO, LUAD, LIHC and KIRP. **(B)** The time-dependent ROC curve of the diagnostic value of TEDC2 in patients with ACC, KIRC, MESO, LUAD, LIHC and KIRP. (*p < 0.05, **p < 0.01, ***p < 0.001).

We further explored the relationship between TEDC2 expression and clinic pathological features in these tumors. The results showed that the expression of TEDC2 in KIRP, KIRC, ACC, LUAD and LIHC was correlated with pathological stage (**Figure 4A**). Moreover, the expression of TEDC2 in LUAD was correlated with age and gender (**Supplementary Figure 3**). The ROC curves were also presented for six tumors whose prognosis was associated with TEDC2 expression (**Figure 4B**), suggesting the diagnostic ability of TEDC2 in these tumors.

**Figure 5:**
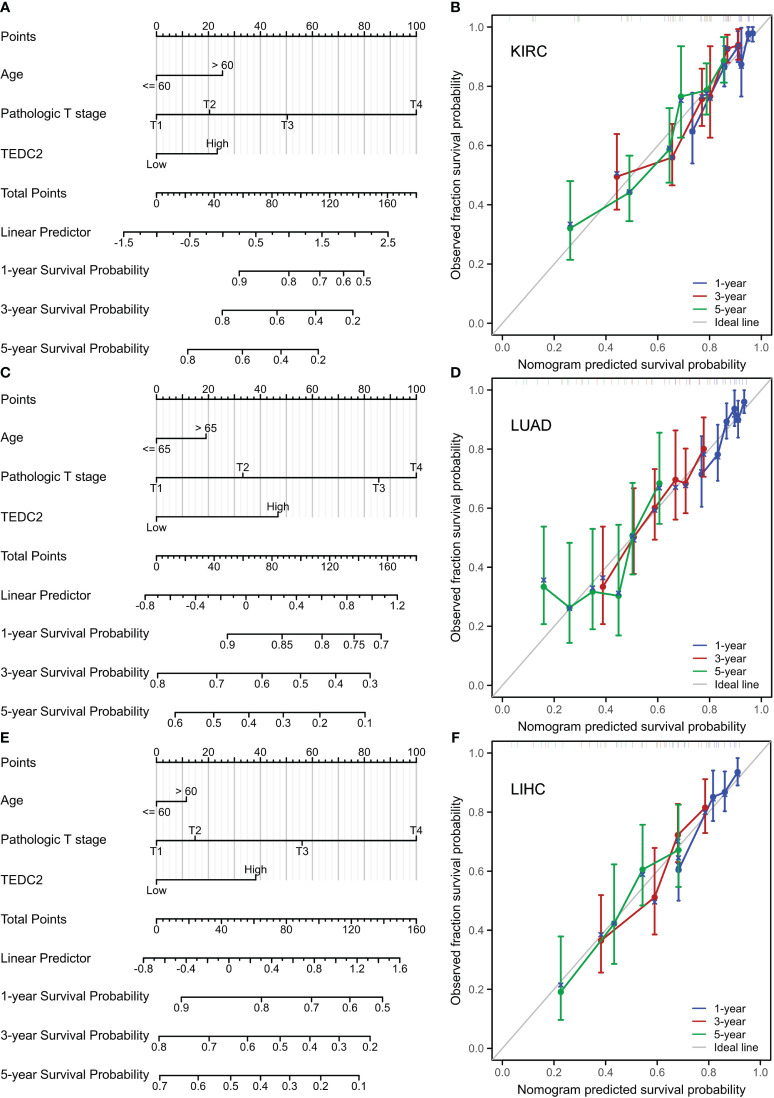
Nomogram models were constructed and evaluated in KIRC, LUAD and LIHC. **(A)** The establishment of a nomogram model combined with the expression of TEDC2 in KIRC. **(B)** Calibration curves were generated to assess the prediction accuracy of the nomograms at 1, 3, and 5 years. **(C)** The establishment of a nomogram model combined with the expression of TEDC2 in LUAD. **(D)** Calibration curves were generated to assess the prediction accuracy of the nomograms at 1, 3, and 5 years in LUAD. **(E)** The establishment of a nomogram model combined with the expression of TEDC2 in LIHC. **(F)** Calibration curves were generated to assess the prediction accuracy of the nomograms at 1, 3, and 5 years in LIHC.

To establish a quantitative prognostic approach for diverse tumor patients, we initially identified, via unvaried Cox analysis, a significant association between the prognosis of multiple tumor patients and the expression of TEDC2, age, and T stage (**Supplementary Tables 2-7**). Subsequently, we integrated these factors into a multivariate Cox model and developed a nomogram model encompassing age, T stage, and TEDC2 expression in three representative tumors (KIRC, LUAD, and LIHC) to validate their prognostic significance. The findings demonstrate that the nomogram model exhibits a high level of accuracy in predicting OS (**Figures 5A, C, E**). We further used calibration curves to evaluate the prediction accuracy of the nomogram model at 1, 3, and 5-years. These results showed that the nomogram models had high accuracy in predicting OS (**Figures 5B, D, F**). Furthermore, in the external validation set, individual risk scores were computed for each patient, and subsequently, they were categorized into high-risk and low-risk groups based on the median. Comparative analysis of survival curves revealed a significantly superior survival rate among patients in the low-risk group as opposed to those in the high-risk group. Additional ROC curves demonstrate that risk signatures possess commendable diagnostic capabilities (**Supplementary Figure 4A, B**).

### Functional enrichment analysis

**Figure 6:**
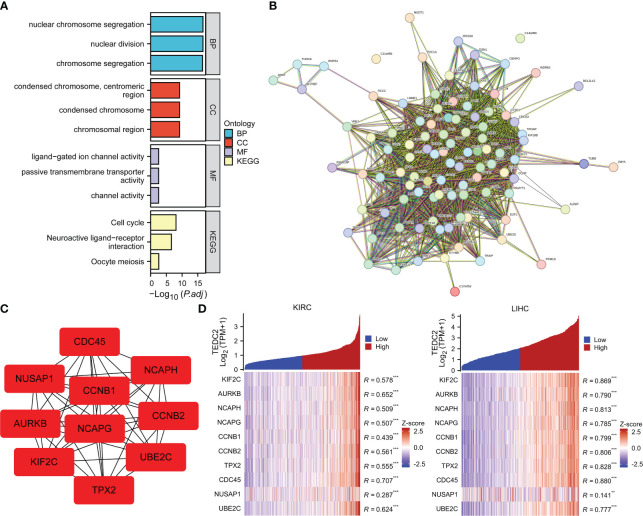
Functional enrichment analysis of TEDC2 related genes. **(A)** GO enrichment (BP, MF and CC) and KEGG pathways analysis based on top 100 TEDC2 related genes. **(B)** PPI network diagram of the top 100 TEDC2 related genes. **(C)** PPI network diagram of the top 10 TEDC2 related genes. **(D)** Correlation analysis of TEDC2 expression and top 10 TEDC2 related genes in KIRC and LIHC in the TCGA database. (**p < 0.01 and ***p < 0.001).

To elucidate the biological function of TEDC2 in tumors, we used GEPIA2 to obtain the top 100 genes with similar expression patterns for TEDC2 in all tumor types. GO enrichment analysis showed that TEDC2 related genes were closely related to nuclear chromosome segregation, nuclear division, condensed chromosome and ligand-gated ion channel activity. KEGG pathway analysis indicated that TEDC2 related genes may participate in to the cell cycle, neuroactive ligand-receptor and oocyte meiosis (**Figure 6A**).

Additionally, a PPI network of the 100 TEDC2 related genes was created from the STRING database (**Figure 6B**), and the top 10 genes were extracted by the cytoHubba plugin in the Cytoscape (version 3.7.1) software (**Figure 6C**). The top 10 genes deeply involved in the regulation of cell proliferation and cell cycle (24–27). Furthermore, we analyzed the correlation between the top 10 genes and TEDC2 expression in KIRC and LIHC. The top 10 genes were plotted in heatmaps. On the right side of heatmaps, significant pairs were identified by Spearman correlation analysis for each of these genes with TEDC2 (**Figure 6D**). The results showed TEDC2 expression correlated positively with these genes, suggesting that TEDC2 may be involved in tumor growth.

In order to elucidate the function of TEDC2, differential gene expression analysis on TEDC2 low (0-30%) and high (70-100%) expression samples was conducted in KIRC, LUAD, and LIHC. KIRC identified 280 genes with significant differential expression, with 222 upregulated and 58 downregulated genes in the high TEDC2 expression group. LUAD identified 755 genes with significant differential expression, with 395 upregulated and 360 downregulated genes in the high TEDC2 expression group. LIHC identified 400 genes with significant differential expression, with 351 upregulated and 59 downregulated genes in the high TEDC2 expression group (**Supplementary Figure 5A**). Subsequently, we used all genes with log2(FC) values for GSEA analysis. Interestingly, a high degree of similarity was found between the enriched gene sets in the three tumors, which included cell cycle checkpoints, cell cycle, cell cycle mitotic and mitotic prometaphase (**Supplementary Figure 5B**).

### Expression of TEDC2 combined with immune infiltration affects overall survival

**Figure 7:**
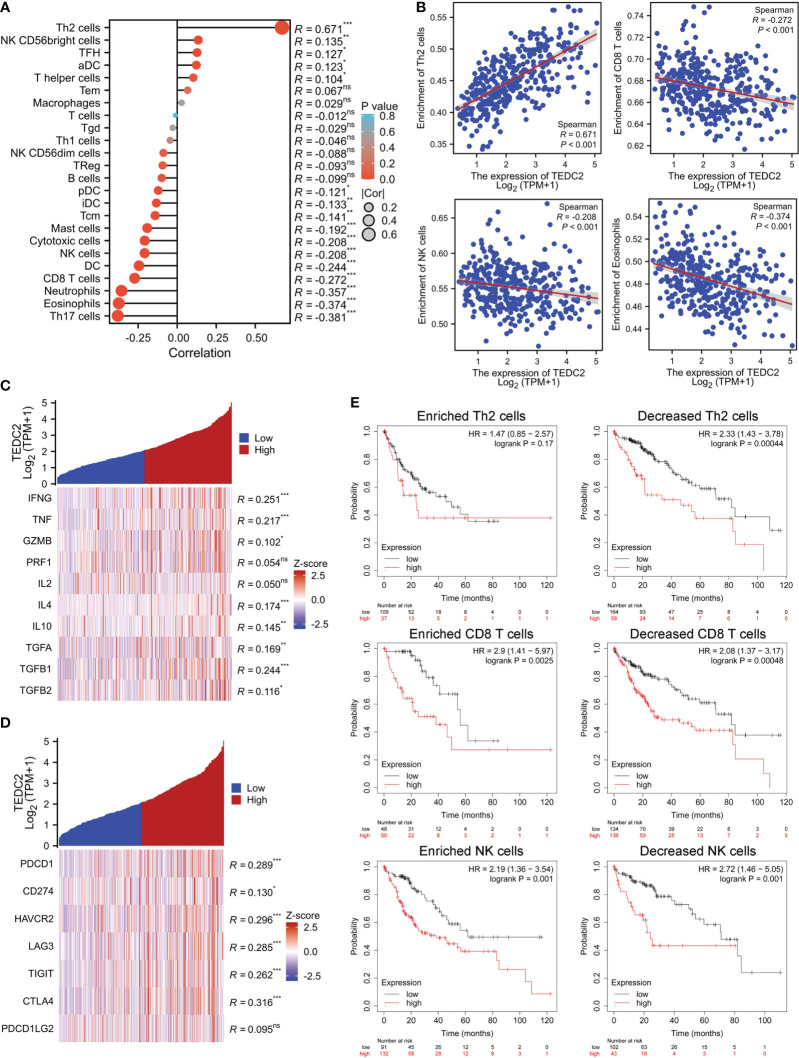
The correlation between immune cell infiltration and TEDC2 expression in LIHC. **(A)** The correlation of TEDC2 expression with the infiltration of different immune cells by ssGSEA algorithm. **(B)** The correlation of TEDC2 expression with eosinophils, NK cells, CD8 T cells and Th2 cells, respectively. **(C)** Correlation analysis of TEDC2 expression and immune related cytokines in LIHC. **(D)** Correlation analysis of TEDC2 expression and immune checkpoint molecules in LIHC. **(E)** Correlations between TEDC2 expression and OS in different immune cell subgroups in LIHC patients were determined by Kaplan–Meier survival plotter. (ns, *p < 0.05, **p < 0.01 and ***p < 0.001).

As we have known that tumor-infiltrated lymphocyte cells play a key role in tumorigenesis and affect the prognosis of tumor patients (28–30). Therefore, we next examine whether TEDC2 is related with the immune infiltration level in specific tumors. We found that TEDC2 expression was negatively correlated with most infiltrated immune cells including CD8 T cells, macrophages, eosinophils, DC cells, cytotoxic cells, and NK cells (**Figure 7A**, **Supplementary Figure 6**). Noteworthy, TEDC2 expression in LIHC was significantly negatively correlated with the enrichment of NK cells (R = −0.208, p < 0.001), CD8 T cells (R = −0.272, p < 0.001) and eosinophils (R = −0.374, P < 0.001). On the contrary, TEDC2 expression was significantly positively correlated with the enrichment of Th2 cells (R = 0.671, p < 0.001) (**Figure 7B**). Considering that TEDC2 may be a potential oncogene in LIHC, the relationship between TEDC2 and various cytokines (IFNG, TNF, GZMB, PRF1, IL2, IL4, IL4, IL10, TGFA, TGFB1, and TGFB2) and immune checkpoints (PDCD1, CD274, TIGIT, LAG3, HAVCR2, CTLA4, and PDCD1LG2) was assessed (**Figures 7C, D**). As a result, we found that the expression of TEDC2 is positively correlated with IFNG, but there is no significant associated with the anti-tumor cytokines GZMB and PRF1. Additionally, TEDC2 expression is positively associated with the immunosuppressive factors IL4, IL10, TGFA and TGFB1. Importantly, the expression levels of TEDC2 had a significant positive correlation with PDCD1, CD274, HAVCR2, LAG3, TIGIT, CTLA4 in LIHC. It is worth noting that we conducted a concise examination of the TEDC2 expression and its prognostic association within four cohorts pertaining to immunotherapy, specifically encompassing two adoptive T cell therapies (**Supplementary Figure 7A, B**) and two immune checkpoint blockade therapies (**Supplementary Figure 7C, D**). We found that the expression of TEDC2 is comparatively diminished in the subset of individuals responding to immunotherapy, and in contrast to those with low TEDC2 expression, patients exhibiting high TEDC2 expression exhibit a markedly reduced survival rate. These results suggested that TEDC2 might mediate the carcinogenic process of tumor by influencing the immunosuppressive microenvironment.

Based on the above results, TEDC2 was associated with immune infiltration of LIHC. We analyzed the effect on tumor survival by combining the expression of TEDC2 and immune cell infiltration. Then, we performed KM plotter analysis of TEDC2 expression in LIHC following CD8 T cells, NK cells and Th2 cells. We found that higher TEDC2 levels in LIHC in enriched CD8 T cells and NK cells had a worse prognosis (**Figure 7E**). These results suggested that immune infiltration might influence the prognosis of tumor with high TEDC2 expression to some extent.

### *In vitro* experimental verification

Based on the above bioinformatics analysis, TEDC2 may be one of the important factors driving the occurrence and development of various tumors. It can activate cell proliferation, induce immune dysfunction, and ultimately lead to poor prognosis. Then, we validated whether knocking down TEDC2 can inhibit the malignant biological behavior of tumor cells in two cell lines, A549 and HepG2.

**Figure 8:**
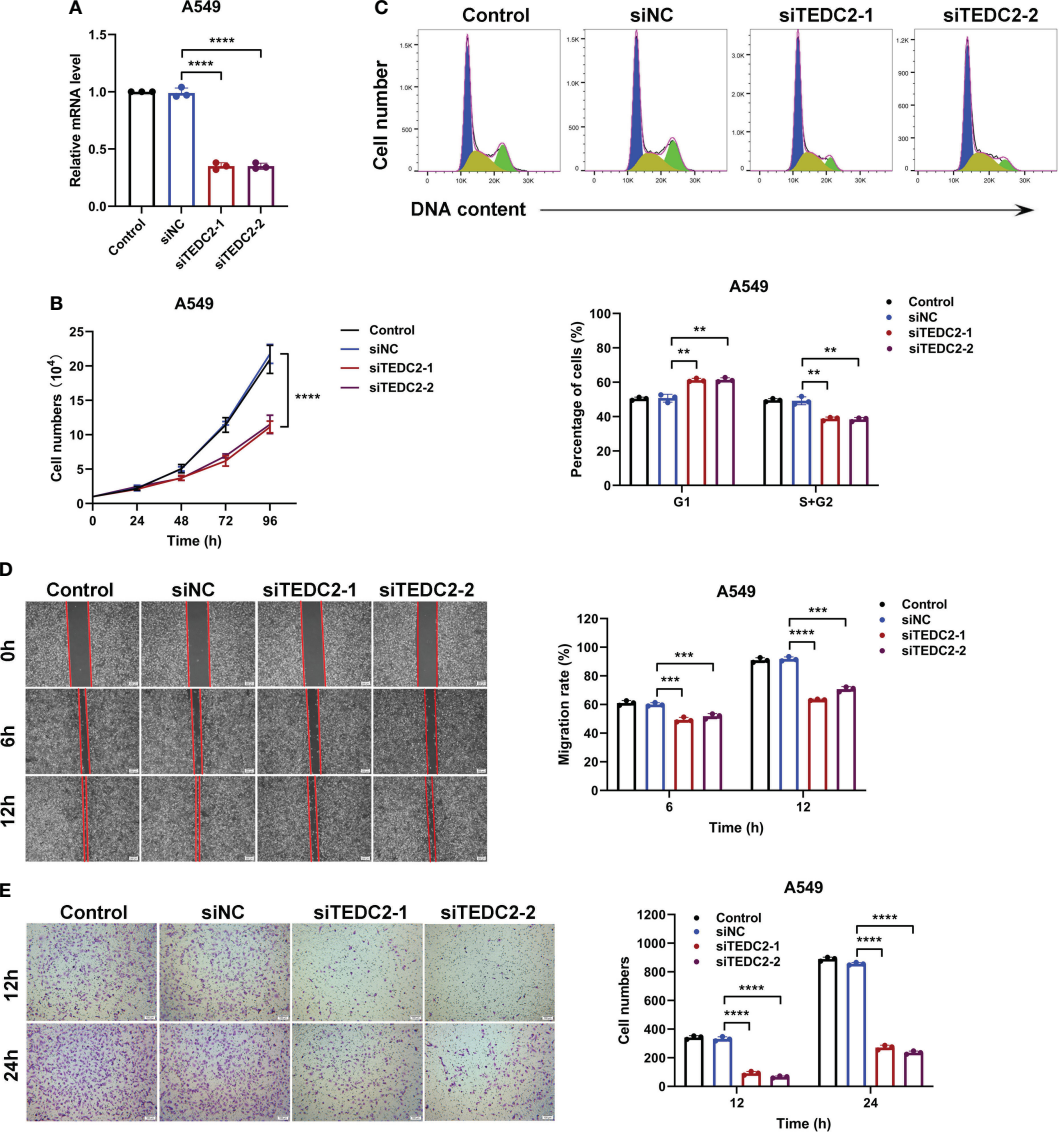
The *in vitro* proliferation and metastasis of A549 cells can be inhibited by the knockout of TEDC2. **(A)** The efficiency of siRNA knockout was evaluated through PCR. **(B)** The survival curves of cells treated with various methods were analyzed. **(C)** Flow cytometry was employed to detect the cell cycle of the Control, siNC, siTEDC2-1, and siTEDC2-2 groups. **(D)** The wound healing experiment was conducted to assess the impact of TEDC2 knockdown on cell migration, and the wound healing ratio was measured following 6 and 12 hours of incubation. **(E)** The Transwell experiment was employed to investigate the influence of TEDC2 knockdown on cell invasion, and the quantitative findings were presented in the Bar chart located on the right. The experiments were conducted in triplicates independently. (**p < 0.01, ***p < 0.001 and ****p < 0.0001).

Based on the HPA database, our findings revealed that the A549 and HepG2 cell exhibited comparatively elevated levels of TEDC2 expression. Furthermore, when compared to the L02 cell line, which represents the normal human liver, the expression of TEDC2 in both A549 and HepG2 cell demonstrated a significant increase (**Supplementary Figure 8A, B**). The knockdown efficiency of siteDC2 was initially assessed using real-time PCR, which revealed that siRNA effectively reduced the mRNA levels of TEDC2 in both A549 and HepG2 cells (**Figure 8A**, **Supplementary Figure 9A**). Additionally, the growth curve analysis demonstrated that the reduction of TEDC2 significantly impeded the proliferation of A549 and HepG2 cells (**Figure 8B**, **Supplementary Figure 9B**). Following this, flow cytometry was employed to examine the impact of TEDC2 knockdown on the cell cycle. The findings of this study indicate that the TEDC2 knockdown group exhibited a significant increase in the proportion of A549 and HepG2 cells in the G1 phase of the cell cycle, accompanied by a significant decrease in the S and G2 phases, when compared to the SINC group (**Figure 8C**, **Supplementary Figure 9C**). These results suggest that the inhibition of TEDC2 can impede the progression of tumor cells through the G1 phase, thereby inhibiting cell proliferation. Additionally, the migration and invasion capabilities of A549 and HepG2 cells were assessed using wound healing and transwell experiments after TEDC2 knockdown. The outcomes revealed a significant reduction in both migration and invasion abilities of the tumor cells following TEDC2 knockdown (**Figures 8D, E** and **Supplementary Figure 9D**).

In conclusion, these findings strongly indicate that TEDC2 assumes a critical role in the etiology and progression of various tumors.

## Discussion

Tumors pose a grave threat to human lives. Despite significant efforts being made to improve tumor diagnosis and treatment, the 5-year overall survival rate for most tumors remains very low (31, 32).. Thus, new methods for diagnosing and treating tumors are urgently needed. The TCGA database utilized multi-omics data to analyze 33 common tumor types, providing an unprecedented opportunity to detect gene functions in different tumor types (33, 34). In recent years, numerous studies have been conducted to identify and characterize pan-cancer molecular biomarkers and their functions, thanks to the advancement of bioinformatics algorithms and databases. In this study, we conducted a thorough analysis by utilizing an open-access database to investigate the prognostic significance and carcinogenic mechanism of TEDC2 across diverse tumor types.

After analyzing data from GEO and TCGA databases, we found that TEDC2 expression was significantly upregulated in various types of tumors, such as ACC, KIRC, LUAD, LIHC, MESO, STAD, and more. To investigate whether TEDC2 could serve as a prognostic marker for tumors, we examined the correlation between TEDC2 expression and the prognosis of different tumor patients. The Kaplan–Meier survival analysis revealed that high TEDC2 expression was associated with adverse survival outcomes in patients with ACC, KIRP, KIRC, LUAD, LIHC, and MESO. These outcomes included OS, PFS, and DSS. Using pan-cancer data, Cox regression analysis identified TEDC2 high expression as an independent risk factor for poor prognosis in tumors. Based on these results, it can be concluded that TEDC2 not only is an overexpressed gene but also serves as a significant prognostic factor for tumor patients.

Based on the above findings, we investigated its downstream mechanisms for carcinogenesis and risk. Firstly, we performed TEDC2 coexpression and functional enrichment analysis. This result indicated that most genes coexpressed with TEDC2 were mainly enriched in cell cycle progression such as nuclear division, chromosome segregation and chromosome condensation, suggesting that these genes could promote tumor growth via accelerating the cell cycle phase. Then, we conducted GSEA databases to analyze the biological functions of TEDC2 in KIRC, LUAD and LIHC. Interestingly, the three tumors showed a high degree of similarity between the enriched gene sets including ECM regulators, cell cycle mitotic and cell cycle checkpoints. To ascertain the precise contribution of TEDC2 in the advancement of tumors, we conducted a comprehensive examination of the biological attributes associated with TEDC2 knockdown in A549 and HepG2 cell lines. Suppression of TEDC2 effectively impedes the cell cycle progression of tumor cells during the G1 phase, consequently impeding cell proliferation. Concurrently, TEDC2 knockdown significantly curtails the migratory and invasive capabilities of tumor cells. These findings further substantiate the potential involvement of TEDC2 in the proliferation of tumor cells.

More and more evidence showed that tumor immune microenvironment plays an important role in tumors. TEDC2 has been identified as a potential oncogenic gene linked to immune infiltration in the tumor microenvironment in two recent studies focusing on hepatocellular carcinoma and laryngeal squamous cell carcinoma (35, 36). In our study, we found that TEDC2 expression was negatively correlated with most infiltrated immune cells, including DC cells, macrophages, CD8 T cells, cytotoxic cells, NK cells and eosinophils, suggesting that TEDC2 might induce tumor immunosuppression. Subsequently, we conducted a detailed analysis in LIHC on the correlation between tumor immune related cytokines and immune checkpoints with TEDC2. The results showed that TEDC2 expression was positively correlated with the immunosuppressive factors IL4, IL10, TGFA, and TGFB1, and with the immune checkpoint molecules such as PDCD1, CTLA4, LAG3, CD274 and HAVCR2. PDCD1 is a negative regulator of T cell function that promotes disease progression in patients with many types of tumors (37, 38). HAVCR2 and LAG3 can work synergistically to promote the exhaustion of effector T cells and inhibit anti-tumor function (39–41). However, the molecular mechanisms underlying TEDC2 and these immune checkpoint molecules are unknown, and require further research. These results suggested that TEDC2 may be involved in modulating the tumor immune microenvironment, suggesting that TEDC2 could be used to develop a new targeted immunotherapy for certain tumors and benefit a large number of tumor patients.

## Conclusions

In conclusion, we found that TEDC2 is associated with prognosis and functions by modulating the immune microenvironment and cell proliferation of various tumors. Admittedly, there are limitations to our study. On the one hand, since all data in this study were obtained from online databases, data heterogeneity is inevitable. On the other hand, some uncommon tumor types have relatively small sample sizes, which can lead to inaccurate results. Finally, this study solely employed bioinformatics methods to analyze the association between TEDC2 and different tumors, and simple experimental verification was conducted. To determine the precise molecular function of TEDC2 in tumor development, additional experiments are required.

## Data availability statement

The raw data supporting the conclusions of this article will be made available by the authors, without undue reservation.

## Author contributions

YL: Data curation, Writing – original draft. JZ: Data curation, Writing – original draft. JS: Data curation, Writing – original draft, Software. YTL: Writing – original draft, Methodology. KP: Writing – original draft, Investigation. CT: Conceptualization, Writing – review & editing. YW: Conceptualization, Project administration, Writing – review & editing.
