# Supplementary material for: A pan-cancer analysis of the prognostic implication and oncogenic role of tubulin epsilon and delta complex 2 (TEDC2) in human tumors
Source: Front Immunol. 2024 Jan 4;14:1272108. doi: 10.3389/fimmu.2023.1272108 (PMC10794491; doi:10.3389/fimmu.2023.1272108)
Supplement: Supplementary file 1 [file DataSheet_1.docx]

**A pan-cancer analysis of the prognostic implication and oncogenic role of tubulin epsilon and delta complex 2 (TEDC2) in human tumours**

Yang Liu^1,5^, Jie Zhu^2,5^, Jing Shen^3,5^, Yuting Lu^4^, Ke Pan^1^, Chuan Tong^4,6*^, Yao Wang^4,6*^

^1^ Faculty of Hepato-Pancreato-Biliary Surgery, the First Medical Centre, Chinese PLA General Hospital, Beijing, China

^2^ Senior Departments of Urology, the Third Medical Centre, Chinese PLA General Hospital, Beijing, China

^3^Department of Endocrinology, the Eighth Medical Center of PLA General Hospital, Beijing, China

^4^ Department of Bio-therapeutic, the First Medical Centre, Chinese PLA General Hospital, Beijing, China

^5^These authors contributed equally: Yang Liu, Jie Zhu and Jing Shen

^6^ These authors jointly supervised this work: Chuan Tong and Yao Wang

* E-mail: tc6636@sina.com; [wangyao301@163.com](mailto:wangyao301@163.com)

**This file includes the following:**

Supplemental Tables S1 to S7

Supplemental Figures S1 and S9

| **Gene name** |  | **Primer sequence** |
| --- | --- | --- |
| TEDC2 | Forward： GCAAATTGCAAGGAGACGC  Reverse： ATTGCAATTGTCGCTGTGC | |
| β-Actin | Forward：GGACTTCGAGCAAGAGATGG  Reverse： AGCACTGTGTTGGCGTACAG | |

Table S1. List of real-time PCR primers.

| **Characteristics** | **Total(N)** | **Univariate analysis** | |  | **Multivariate analysis** | |
| --- | --- | --- | --- | --- | --- | --- |
|  |  | **Hazard ratio (95% CI)** | **P value** |  | **Hazard ratio (95% CI)** | **P value** |
| Age  (<= 60/> 60) | 373  (177/196) | 1.205 (0.850 - 1.708) | 0.295 |  |  |  |
| Gender (Female/Male) | 373 (121/252) | 0.793 (0.557 - 1.130) | 0.200 |  |  |  |
| Pathologic stage (SI&SII/SIII&SIV) | 349  (259/90) | 2.504 (1.727 - 3.631) | **< 0.001** |  | 1.985 (1.327 - 2.968) | **< 0.001** |
| Tumor status (Free/With tumor) | 354  (202/152) | 2.317 (1.590 - 3.376) | **< 0.001** |  | 1.776 (1.187 - 2.657) | **0.005** |
| TEDC2  (Low/High) | 373  (187/186) | 2.026 (1.421 - 2.888) | **< 0.001** |  | 1.659 (1.115 - 2.469) | **0.013** |

Table S2. Univariate and multivariate COX regression in patients with LIHC.

| **Characteristics** | **Total(N)** | **Univariate analysis** | |  | **Multivariate analysis** | |
| --- | --- | --- | --- | --- | --- | --- |
|  |  | **Hazard ratio (95% CI)** | **P value** |  | **Hazard ratio (95% CI)** | **P value** |
| Age  (<= 65/> 65) | 520  (267/263) | 1.216 (0.910 - 1.625) | 0.186 |  |  |  |
| Gender (Female/Male) | 530  (283/247) | 1.087 (0.816 - 1.448) | 0.569 |  |  |  |
| Pathologic stage (SI&SII/SIII&SIV) | 522  (415/107) | 2.710 (1.994 - 3.685) | **< 0.001** |  | 2.343 (1.629 - 3.368) | **< 0.001** |
| Residual tumor (R0/R1&R2) | 365  (349/16) | 3.913 (2.188 - 6.997) | **< 0.001** |  | 2.976 (1.636 - 5.412) | **< 0.001** |
| TEDC2  (Low/High) | 530  (266/264) | 1.681 (1.256 - 2.251) | **< 0.001** |  | 1.449 (1.034 - 2.030) | **0.031** |

Table S3. Univariate and multivariate COX regression in patients with LUAD.

| **Characteristics** | **Total(N)** | **Univariate analysis** | |  | **Multivariate analysis** | |
| --- | --- | --- | --- | --- | --- | --- |
|  |  | **Hazard ratio (95% CI)** | **P value** |  | **Hazard ratio (95% CI)** | **P value** |
| Age  (<= 65/> 65) | 86  (46/40) | 1.325 (0.826 - 2.125) | 0.243 |  |  |  |
| Gender (Female/Male) | 86  (16/70) | 0.888 (0.494 - 1.595) | 0.691 |  |  |  |
| Pathologic stage (SI&SII/SIII&SIV) | 86  (26/60) | 0.993 (0.588 - 1.675) | 0.978 |  |  |  |
| Residual tumor (R0/R1&R2) | 35  (17/18) | 1.086 (0.522 - 2.259) | 0.824 |  |  |  |
| TEDC2  (Low/High) | 86  (42/44) | 2.739 (1.679 - 4.468) | **< 0.001** |  | 2.739 (1.679 - 4.468) | **< 0.001** |

| **Characteristics** | **Total(N)** | **Univariate analysis** | |  | **Multivariate analysis** | |
| --- | --- | --- | --- | --- | --- | --- |
|  |  | **Hazard ratio (95% CI)** | **P value** |  | **Hazard ratio (95% CI)** | **P value** |
| Age  (<= 60/> 60) | 288  (135/153) | 0.969 (0.533 - 1.762) | 0.917 |  |  |  |
| Gender (Female/Male) | 290  (77/213) | 0.630 (0.327 - 1.213) | 0.167 |  |  |  |
| Pathologic stage (SI&SII/SIII&SIV) | 260  (193/67) | 6.261 (3.252 - 12.052) | **< 0.001** |  | 3.148 (0.702 - 14.109) | 0.134 |
| Pathologic N stage  (N0/N1&N2) | 78  (50/28) | 5.220 (2.153 - 12.658) | **< 0.001** |  | 2.624 (0.828 - 8.312) | 0.101 |
| TEDC2  (Low/High) | 290  (144/146) | 1.962 (1.057 - 3.640) | **0.033** |  | 0.878 (0.362 - 2.127) | 0.773 |

Table S4. Univariate and multivariate COX regression in patients with MESO.

Table S5. Univariate and multivariate COX regression in patients with KIRP.

| **Characteristics** | **Total(N)** | **Univariate analysis** | |  | **Multivariate analysis** | |
| --- | --- | --- | --- | --- | --- | --- |
|  |  | **Hazard ratio (95% CI)** | **P value** |  | **Hazard ratio (95% CI)** | **P value** |
| Age  (<= 60/> 60) | 541  (269/272) | 1.791 (1.319 - 2.432) | **< 0.001** |  | 1.630 (1.196 - 2.222) | **0.002** |
| Gender (Female/Male) | 541  (187/354) | 0.924 (0.679 - 1.257) | 0.613 |  |  |  |
| Pathologic stage (SI&SII/SIII&SIV) | 538  (332/206) | 3.910 (2.852 - 5.360) | **< 0.001** |  | 2.942 (2.103 - 4.115) | **< 0.001** |
| Histologic grade (G1&G2/G3&G4) | 533  (250/283) | 2.665 (1.898 - 3.743) | **< 0.001** |  | 1.656 (1.151 - 2.382) | **0.007** |
| TEDC2  (Low/High) | 541(270/271) | 1.843 (1.358 - 2.500) | **< 0.001** |  | 1.486 (1.085 - 2.036) | **0.014** |

| **Characteristics** | **Total(N)** | **Univariate analysis** | |  | **Multivariate analysis** | |
| --- | --- | --- | --- | --- | --- | --- |
|  |  | **Hazard ratio (95% CI)** | **P value** |  | **Hazard ratio (95% CI)** | **P value** |
| Age  (<= 50/> 50) | 79  (41/38) | 1.799 (0.846 - 3.824) | 0.127 |  |  |  |
| Gender (Female/Male) | 79  (48/31) | 1.001 (0.469 - 2.137) | 0.999 |  |  |  |
| Pathologic stage (SI&SII/SIII&SIV) | 77  (46/31) | 6.476 (2.706 - 15.498) | **< 0.001** |  | 3.208 (1.143 - 9.004) | **0.027** |
| TEDC2  (Low/High) | 79(39/40) | 7.129 (2.838 - 17.907) | **< 0.001** |  | 5.064 (1.541 - 16.637) | **0.008** |

Table S6. Univariate and multivariate COX regression in patients with KIRC.

Table S7. Univariate and multivariate COX regression in patients with ACC.


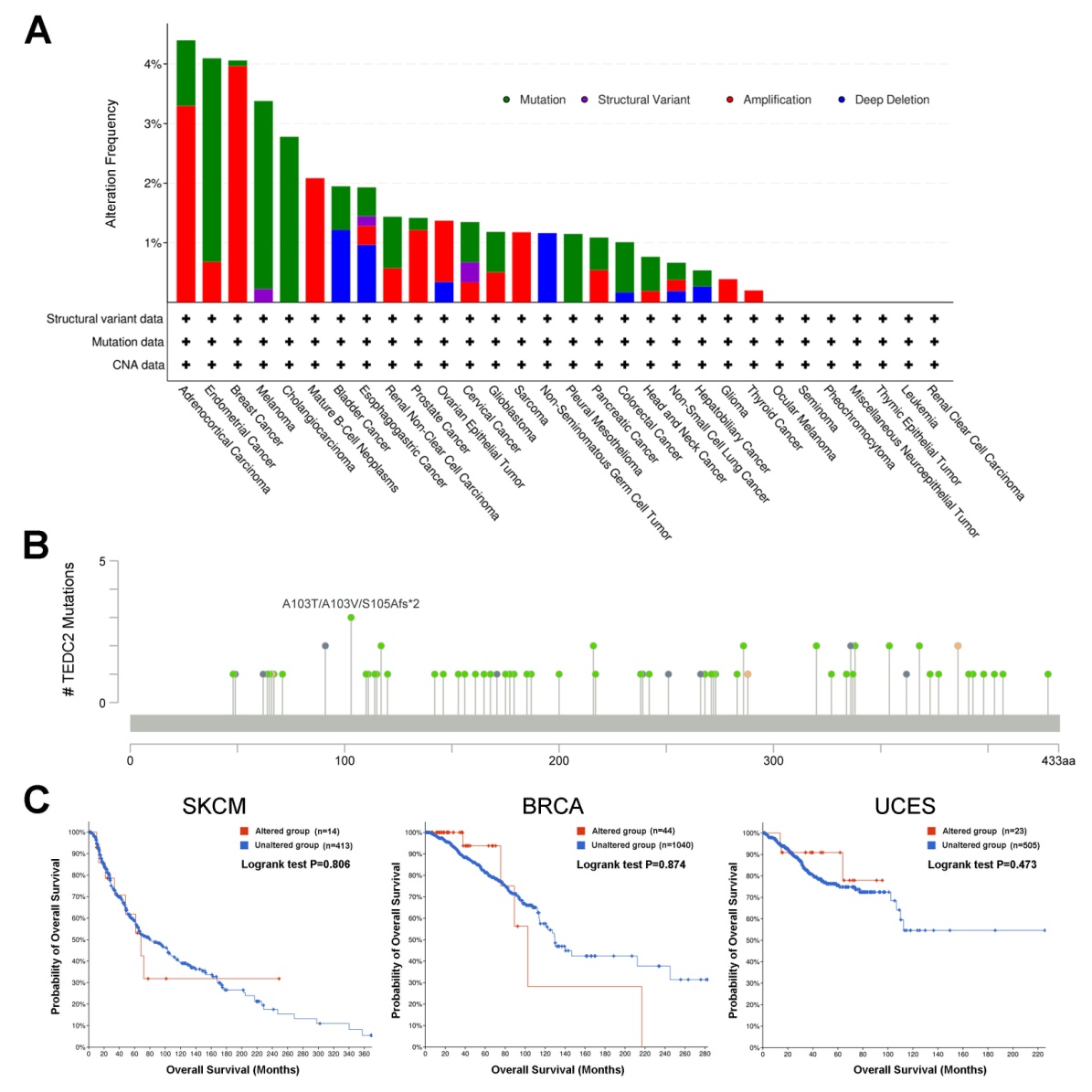


Fig.S1 TEDC2 genetic alteration in various tumor types of TCGA. (A) Alterations summary of TEDC2 in pan-cancer datasets. (B) The mutation types, number, and sites of the TEDC2 genetic alterations. (C) The correlations between TEDC2 amplification status and overall survival of SKCM, BRCA, UCES were analyzed by cBioPortal.


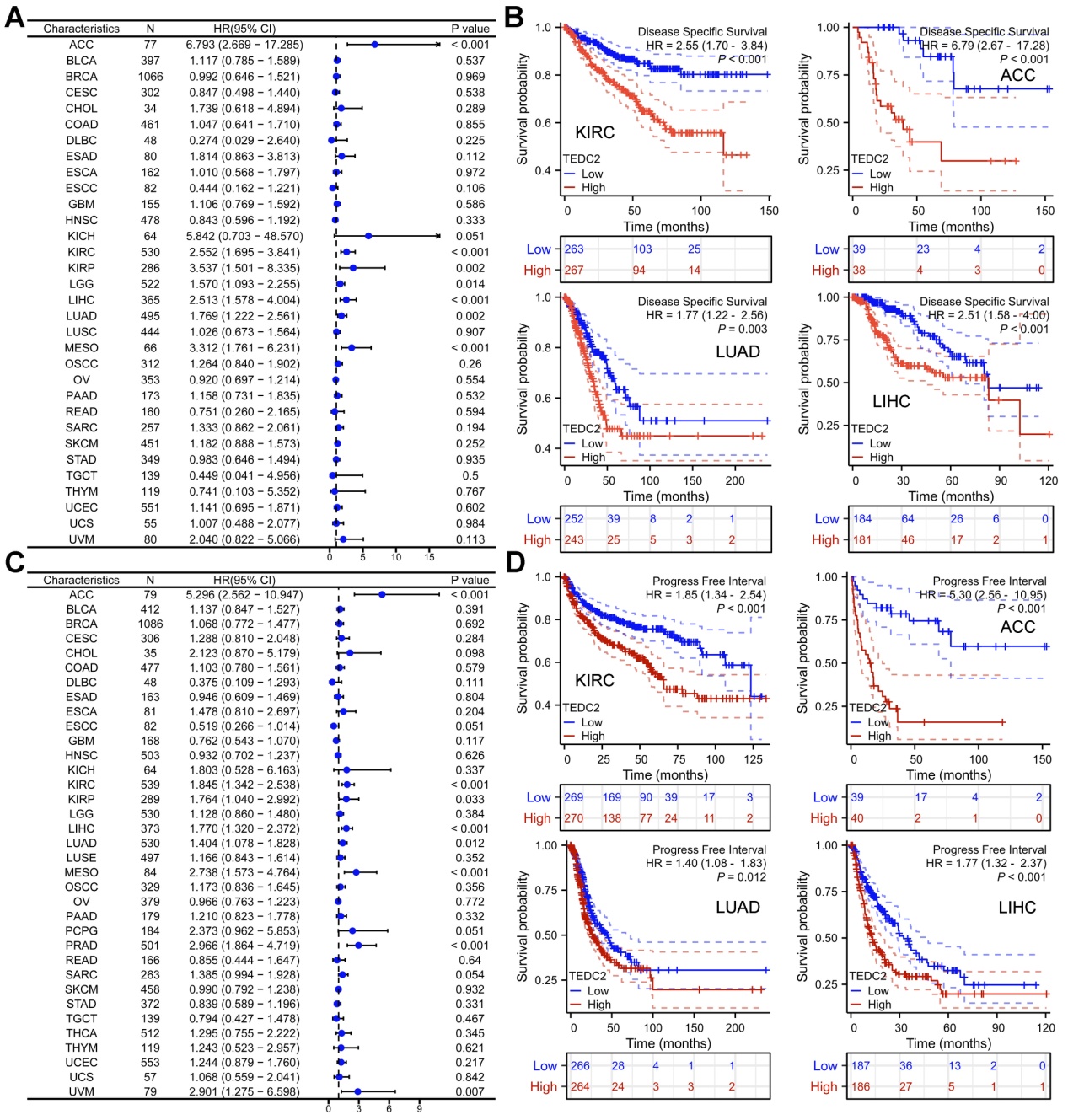


Fig.S2 The association between TEDC2 expression and DSS and PFI in pan-cancer. (A) The effects of TEDC2 expression on DSS in pan-cancer were exhibited by a forest plot. (B) Effects of TEDC2 expression on DSS in KIRC, ACC, LUSC and LIHC, respectively. (C) The effects of TEDC2 expression on PFI in pan-cancer were exhibited by a forest plot. (D) Effects of TEDC2 expression on PFI in KIRC, ACC, LUSC and LIHC, respectively.


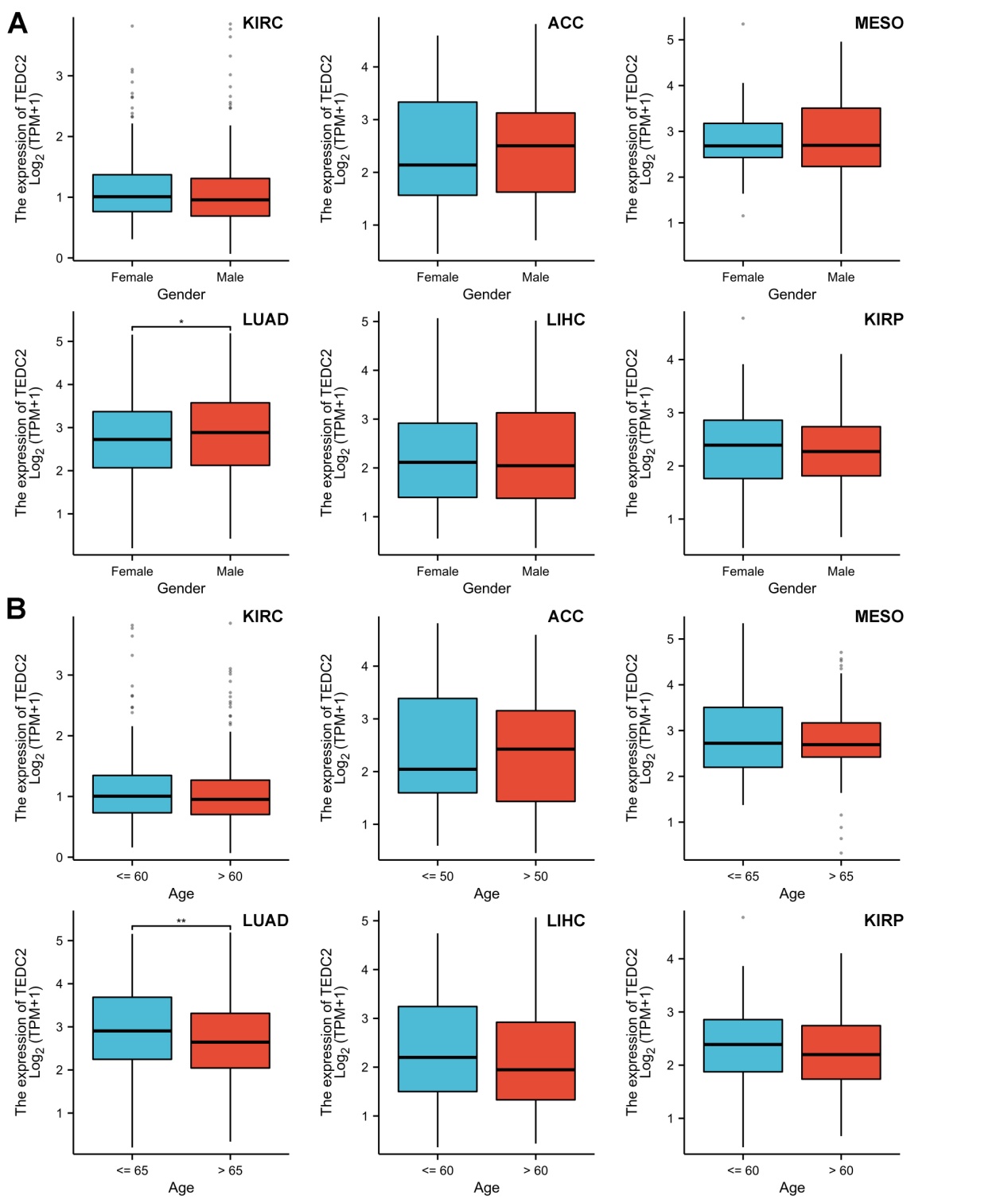


Fig.S3 The correlation between TEDC2 expression and clinic pathological parameters. (A) TEDC2 expression was correlated with gender in KIRC, ACC, MESO, LUAD, LIHC and KIRP. (B) TEDC2 expression was correlated with age in KIRC, ACC, MESO, LUAD, LIHC and KIRP.


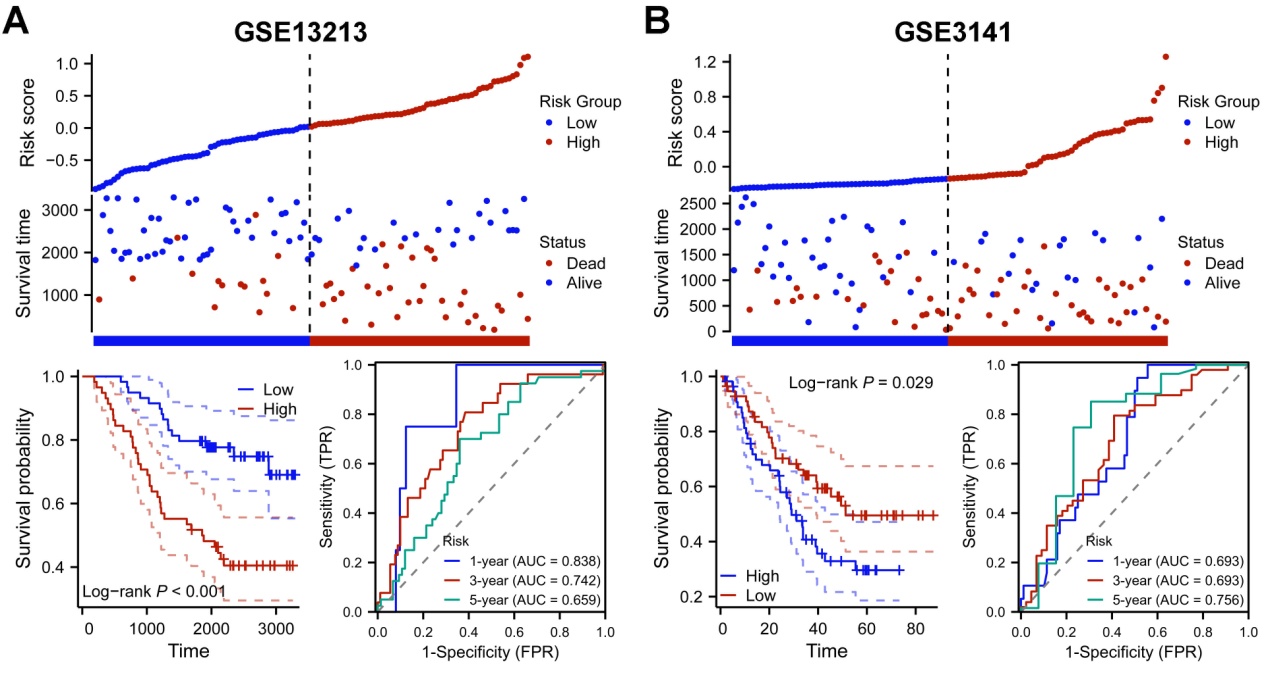


Fig.S4 (A, B) The distribution of risk scores and corresponding survival status, as well as conducts survival analysis and evaluates the receiver operating characteristic (ROC) curve in two external validation datasets.


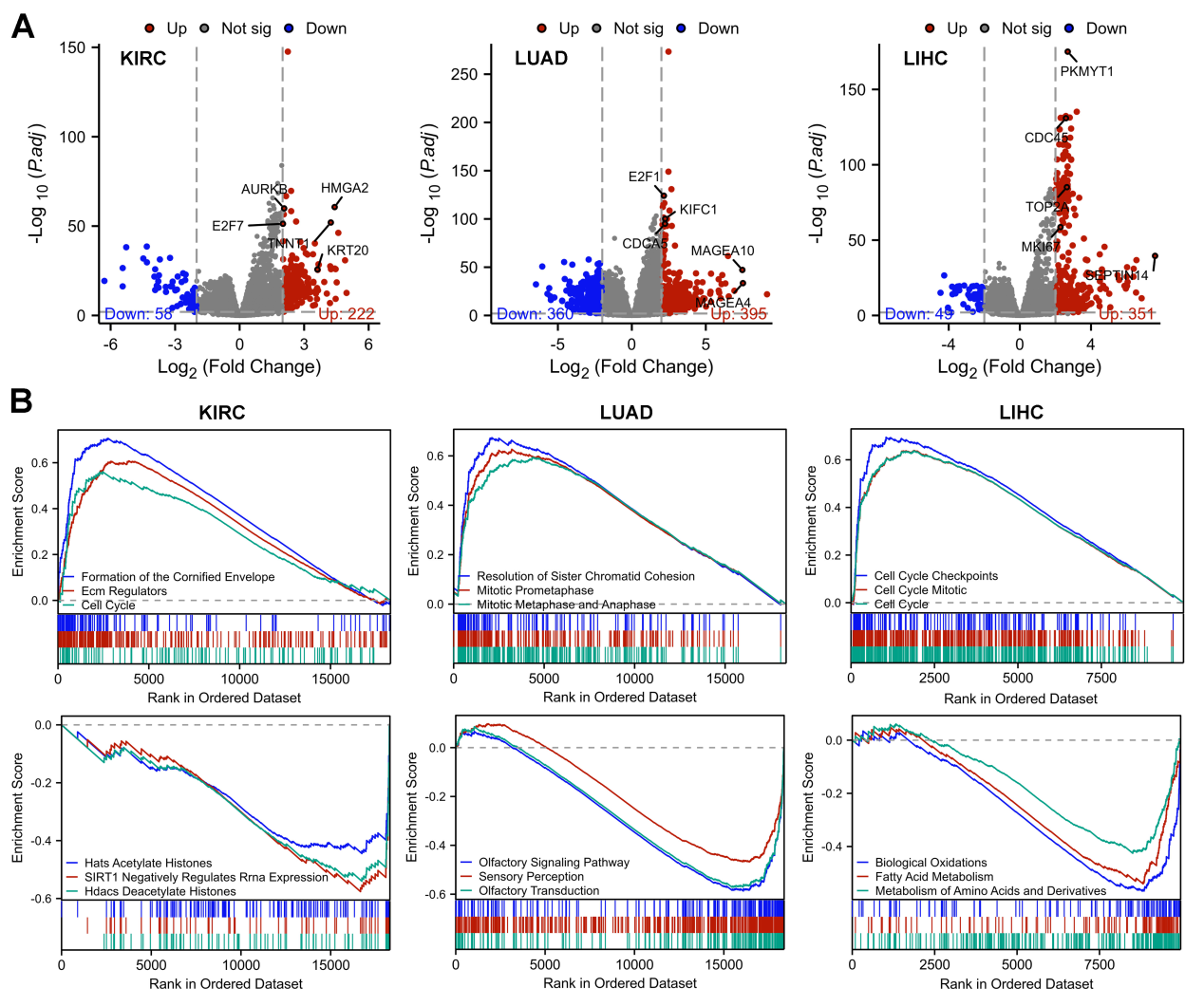


Fig.S5 Differential gene expression in KIRC, LUAD and LIHC patients with low- and high- expression of TEDC2. (A) Volcanic plots showed differential gene expression in patients with KIRC, LUAD, and LIHC. (B) GSEA analysis of differentially expressed genes of KIRC, LUAD and LIHC.


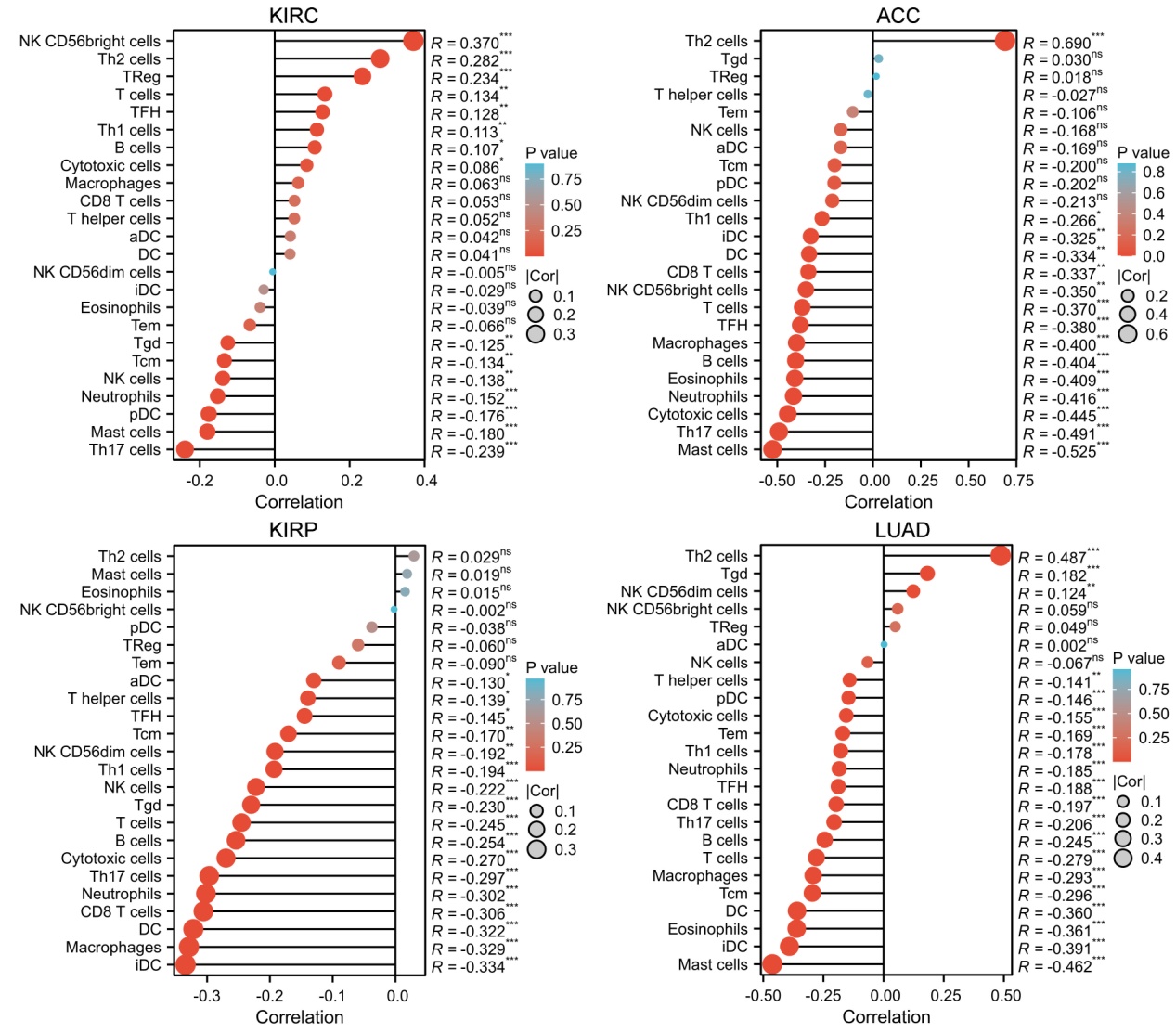
 Fig.S6 The correlation of TEDC2 expression with the infiltration of different immune cells in KIRC, ACC,KIRP and LUAD by ssGSEA algorithm.


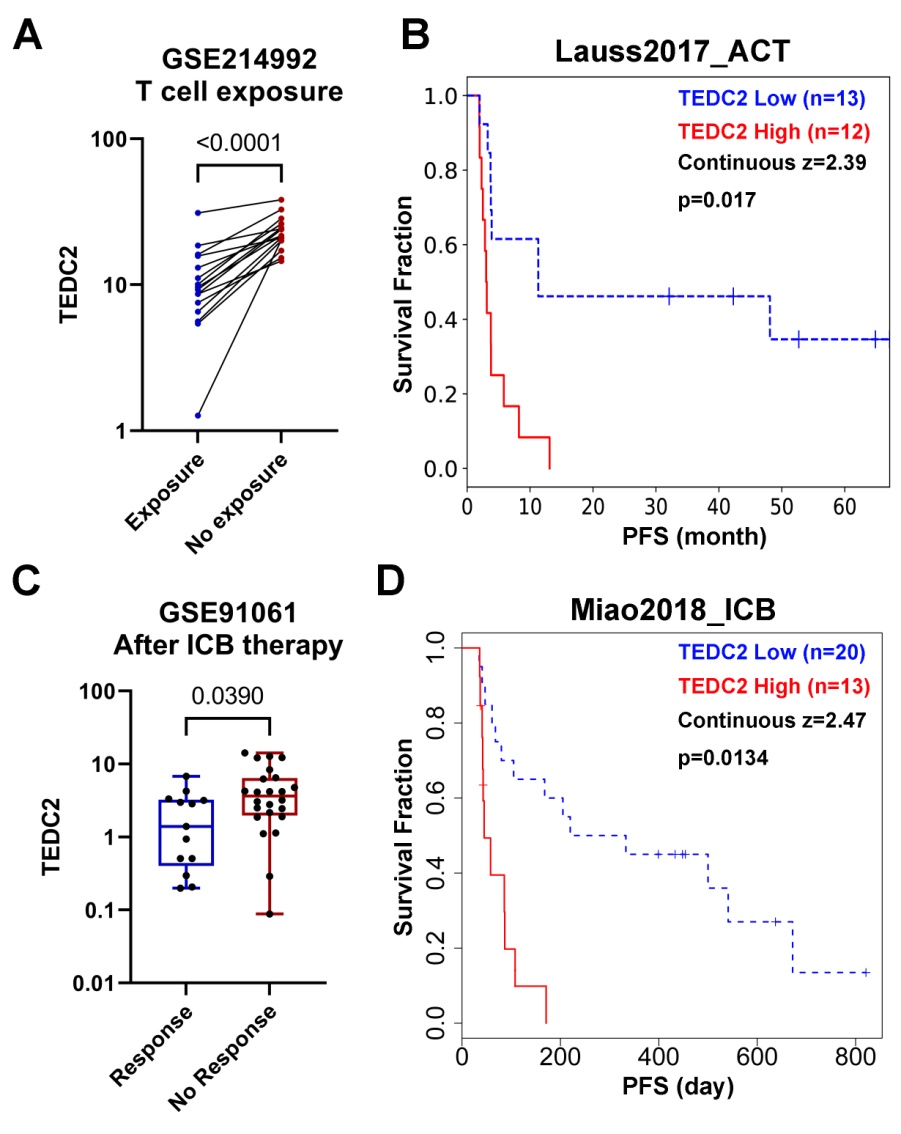


Fig.S7 The relationship between TEDC2 and treatment response in four immunotherapy cohorts(A, B two adoptive T cell therapy, C, D two immune checkpoint blockade therapy).


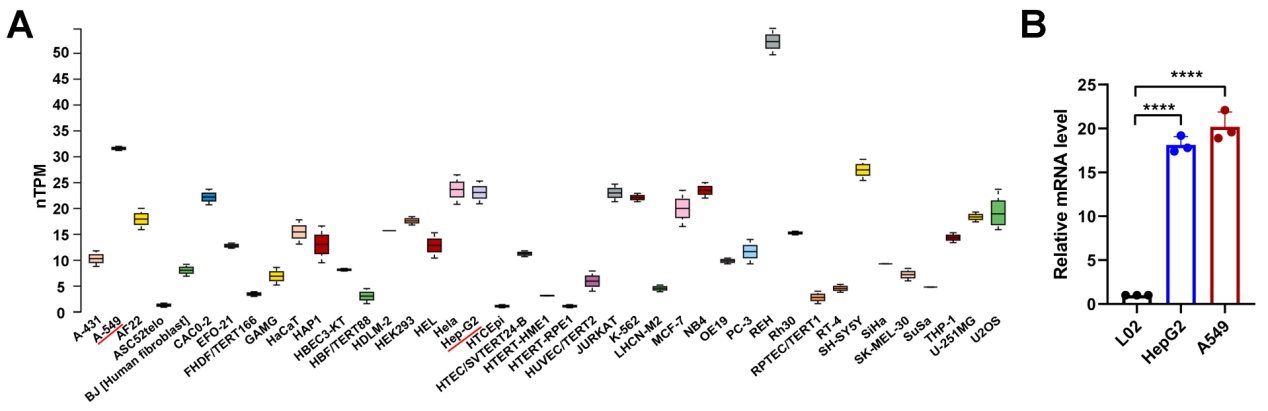
Fig.S8 The mRNA expression of TEDC2 in cell lines. (A) TEDC2 expression in cell lines based on the HPA database. (B) The expression of TEDC in cell lines was detected using real-time -PCR. (ns, *p < 0.05, **p < 0.01, ***p < 0.001 and ****p < 0.0001)


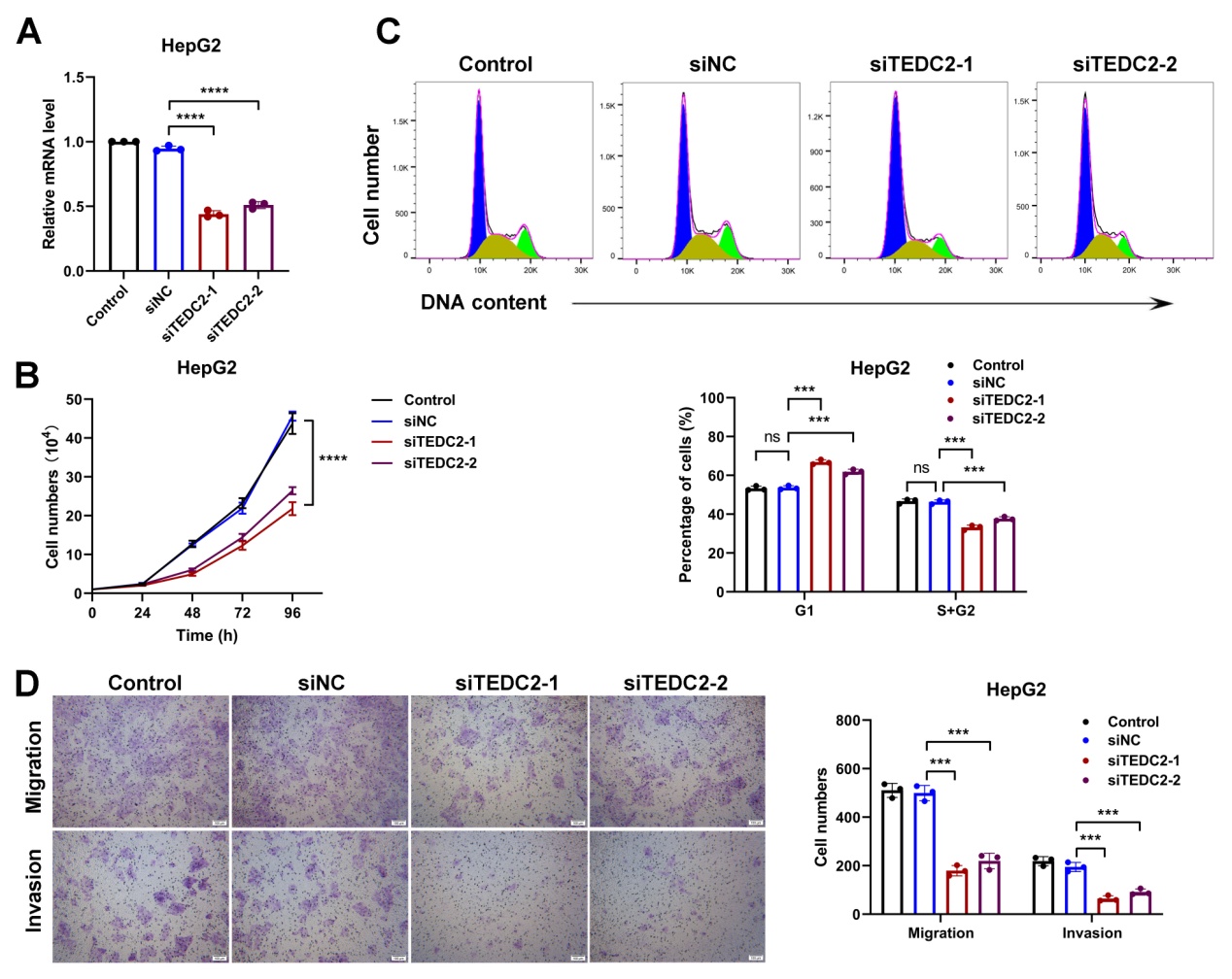


Fig.S9 The in vitro proliferation and metastasis of HepG2 cells can be inhibited by the knockout of TEDC2. (A) The efficiency of siRNA knockout was evaluated through PCR. (B) The survival curves of cells treated with various methods were analyzed. (C) Flow cytometry was employed to detect the cell cycle of the Control, siNC, siTEDC2-1, and siTEDC2-2 groups, and the quantitative findings were presented in the Bar chart located on the bottom. (D) The Transwell experiment was employed to investigate the influence of TEDC2 knockdown on cell migration and invasion, and the cell number was measured following 72 hours of incubation. The experiments were conducted in triplicates independently. (ns, *p < 0.05, **p < 0.01, ***p < 0.001 and ****p < 0.0001)
