# Supplementary material for: A pan-cancer analysis of the prognostic implication and oncogenic role of tubulin epsilon and delta complex 2 (TEDC2) in human tumors
Source: Front Immunol. 2024 Jan 4;14:1272108. doi: 10.3389/fimmu.2023.1272108 (PMC10794491; doi:10.3389/fimmu.2023.1272108)
Supplement: Supplementary file 2 [file DataSheet_2.docx]

https://www.jianguoyun.com/p/DTO46poQk87sCxiP15MFIAA
